# Development of a Blocking Primer to Inhibit the PCR Amplification of the 18S rDNA Sequences of *Litopenaeus vannamei* and Its Efficacy in *Crassostrea hongkongensis*

**DOI:** 10.3389/fmicb.2019.00830

**Published:** 2019-04-23

**Authors:** Cong Liu, Ren-Jie Qi, Jing-Zhe Jiang, Ming-Qing Zhang, Jiang-Yong Wang

**Affiliations:** ^1^Key Laboratory of Aquatic Product Processing, Ministry of Agriculture and Rural Affairs, South China Sea Fisheries Research Institute, Chinese Academy of Fishery Sciences, Guangzhou, China; ^2^College of Fisheries and Life Science, Shanghai Ocean University, Shanghai, China; ^3^College of Fisheries, Tianjin Agricultural University, Tianjin, China; ^4^Shenzhen Base of South China Sea Fisheries Research Institute, Chinese Academy of Fishery Sciences, Shenzhen, China; ^5^School of Life Science, Guangzhou University, Guangzhou, China

**Keywords:** blocking primer, PCR, eukaryotic microorganisms diversity, high-throughput sequencing, shrimp, oyster

## Abstract

Diversity analyses of the eukaryotic microorganisms in the gut of marine animals is hampered by the presence of host DNA in the samples. PCR amplification of rRNA genes of eukaryotic microorganisms is inefficient with universal primers targeting 18S rRNA gene when the host DNA is dominant. In this study, we designed several blocking primers to inhibit PCR amplification of rRNA genes of the shrimp *Litopenaeus vannamei*, and tested their efficacy on the oyster *Crassostrea hongkongensis*. We first compared the intensity of PCR product bands obtained with and without the blocking primers. Then, one primer was selected for further verification using high-throughput sequencing. Our results showed that X-BP2-DPO was the most effective blocking primer in suppressing the host 18S amplification compared to nine other candidates. The inhibition rate was 99% for the amplification of shrimp rDNA, and 17% for the amplification of oyster rDNA. The concentration of the blocking primer in the PCR mixture was an important factor to be considered in the experimental design. The development of blocking primers provided a valid method to study the composition and characteristics of eukaryotic microorganisms in shrimp gut for a better understanding of its diets.

## HIGHLIGHTS

- This is the first study of eukaryotic microbiota of shrimp and oyster.
- An effective and specific blocking primer was developed to block the 18S amplification of the host DNA in shrimp (*L. vannamei*).
- The inhibition rate of X-BP2-DPO for the amplification of shrimp rDNA was as high as 99%.

## Introduction

A major focus of ecology is understanding trophic relationships and energy flows in natural environments, associated food web dynamics and changes in food webs due to disturbance, environmental change, invasions and extinctions. Predator-prey interactions are often assessed by examining eukaryotic microbes in gut contents (Aguilar et al., 2017). Traditionally, the identification of gut content species has been conducted using morphological methods (Paquin et al., 2014; Harms-Tuohy et al., 2016; Aguilar et al., 2017). However, characterizing the interaction between the host and the non-host via such methods may introduce a number of uncertainties that increase the difficulty of determining the complex diversity or heterogeneity of eukaryotes in animals (Bailey et al., 2010).

As a result, several methods for analyzing the diversity of eukaryotic microorganisms in the gut of marine animals have been tested. Of them, DNA-based methods are perhaps the most promising ones, and are now established as powerful tools for studying the diversity of eukaryotes (Symondson, 2002; King et al., 2008; Harms-Tuohy et al., 2016). The amplification of the 18S rRNA genes (rDNA) of eukaryotes by the polymerase chain reaction (PCR) makes it easier to understand the species composition of samples (Maslov et al., 2013). However, PCR-based studies typically employ several cycles and target short amplicon sizes, thereby allowing co-amplification of certain quantities of the host DNA in the samples (Martin and Rygiewicz, 2005; Vestheim et al., 2005). Mostly, the species diversity that could be detected by universal primers in samples is often compromised by high concentrations of the host DNA templates (Vestheim and Jarman, 2008). If the sample contains the host sequences at relatively high concentrations, then the less concentrated sequences of other eukaryotes are often not amplified as PCR favors the dominant DNA types (Jarman et al., 2004; Deagle et al., 2005; Belda et al., 2017).

A common method to circumvent this problem is to design species-specific primers for detecting the species of interest in the gut of animals (Agusti et al., 2003; Nejstgaard et al., 2003; Vestheim et al., 2005). This is, however, not suitable if the potential range of the target species is large or unknown, as it is impossible to design hundreds of species-specific primers in advance. Restriction enzymes have been applied in PCR amplification to remove host DNA (Blankenship and Yayanos, 2005; Blankenship and Levin, 2007; Flaherty et al., 2018). It requires a unique cutting site in the host sequence which is often difficult to find. Furthermore, it will not work if the PCR has failed to amplify any non-host DNA when the host DNA has dominated the PCR completely. Green and Minz (2005) applied a method, “Suicide Polymerase Endonuclease Restriction,” to cut only the target DNA in the mix prior to the investigation for microbial diversity using PCR. However, this method still requires several additional handling steps and has apparently not achieved wide acceptance.

A new method is to design a blocking primer which can block the amplification of host DNA in a complex sample for the detection of eukaryotic species of interest (Seyama et al., 1992; Vestheim and Jarman, 2008; Craig et al., 2014). Blocking primers preferentially bind to the DNA of which amplification is to be avoided, i.e., the host DNA. They are synthesized like conventional amplification primers, but modified with the addition of a C3 spacer at the 3′ end, resulting in complete inhibition of the enzymatic elongation of the primer. In PCR, blocking primers can either compete directly with the amplification primers (annealing-inhibiting blocking primers) or prevent elongation by binding onto the fragment in between the two amplification primers (elongation arrest blocking primers) (Boessenkool et al., 2012). Su et al. (2017) enhanced the detection of prey-specific DNA sequences via high-throughput sequencing using blocking primers, which has shown that different kinds of fish consumed a wide variety of prey. Boessenkool et al. (2012) applied a human-specific blocking primer to study the diversity of endogenous DNA sequences. Powell et al. (2012) has reported the development of a new blocking primer that allows PCR amplification of bacterial DNA in the presence of algal chloroplast DNA. Anneal-inhibiting blocking primers and peptide-nucleic acid (PNA) oligonucleotide blockers have been developed to reduce the amplification of mosquito 18S rRNA gene sequences (Belda et al., 2017). Nevertheless, there are few studies on host inhibition primers in marine animals, especially in invertebrates, which could help understand their eukaryotic microbiota (Clerissi et al., 2018).

Considering that shrimps and oysters are among the most important and popular aquaculture species in the world, the development of primers blocking their rRNA genes will aid to a better understanding of their diets and even allow the detection of parasites and pathogens in these animals. In this study, we designed a blocking primer capable of inhibiting the amplification of shrimp host DNA, tested its optimal concentration for the suppression of 18S rDNA amplification, and also validated its effectiveness in oysters.

## Materials and Methods

### Design of Blocking Primer

From the NCBI web site^1^, we retrieved 18S rDNA sequences from shrimp, oyster, and protist which are considered to be common and representative species in aquaculture ponds (Ucko et al., 1989; Shekhar, 1997; Purivirojkul and Khidprasert, 2009; Hamoutene et al., 2015). We performed multi-sequence alignments of the 18S rDNA sequences (Supplementary Figure S1), and defined conserved regions as targets to our blocking primers. These regions correspond to priming regions targeting the V9 of 18S rRNA genes, referred to the Earth Microbiome Project^2^ (Amaral-Zettler et al., 2009; Albaina et al., 2016). The universal primers are as follows: 1391f (forward): 5′-GTACACACCGCCCGTC-3′; and EukBr(reverse): 5′-TGATCCTTCTGCAGGTTCACCTAC-3′. We followed two strategies aiming to block the host sequence:

(a) The blocking primer was modified with a C3 spacer at the 3′-end.
(b) The blocking primer had an internal modification of five deoxyinosine molecules (44444), in addition to a C3 spacer added at the 3′-end.

The C3 spacer (3 hydrocarbons, 1-dimethoxytrityloxy-propanediol-3-succinoyl-long chain alkylamino) is a standard primer modification available from most suppliers of custom oligonucleotides (Tan and Liu, 2018). Adding this modification to the 3′-end of an oligonucleotide prevents elongation during PCR, without noticeably influencing its annealing properties (Chun et al., 2007; Vestheim and Jarman, 2008). Dual priming oligonucleotide (DPO) contains two separate priming regions joined by a polydeoxyinosine linker, which is used to decrease the melting temperature of long primers and avoiding mispriming and primer-dimer and hairpin formation (Wu et al., 1991; Vestheim and Jarman, 2008).

The blocking primer intended to inhibit amplification of shrimp DNA was mainly designed based on the differences among the 18S rDNA sequences of protists, and those of shrimp. We selected the blocking sites which are unique in shrimp sequences, based on the alignments of the 18S rDNA sequences of the various species of eukaryotes, including shrimp, oyster, algae (Rhodophyta, Chlorophyta), and protozoan (Platyhelminthes, Endomyxa, Arthropoda) in Supplementary Figure S1. The details of the accession numbers of the 18S rDNA sequences of different eukaryotic species (with annotation at Phylum level) are shown in Supplementary Table S1. The 5′ end of the blocking primer starts with the common section, which is consistent with the forward universal primer either partially or completely, and extends to a position where there is a clear difference between the 18S rDNA sequences of shrimp and other eukaryotes. We found the sequence of algae is quite different from that of shrimp and protist after comparing their 18S rDNA sequences (Supplementary Figure S1A).

In this study, 10 blocking primers were designed according to alignments of the 18S rDNA sequences of several eukaryotes such as shrimp, oyster, eukaryotic algae, and protist. The first six primers were designed against the shrimp host and the other four were designed against the oyster host (Supplementary Table S2).

### Sample Collection and DNA Extraction

Samples of *Litopenaeus vannamei* and *Crassostrea hongkongensis* were caught in October, 2017 from a pond at Fenzhou Village, Doumen District, Zhuhai City, Guangdong Province where shrimp and shellfish polyculture farming is carried out. The gut of *L. vannamei* and *C. hongkongensis* were immediately collected under aseptic conditions and preserved in a DNA storing reagent (GT21234, Huayueyang, Beijing). Three groups of shrimp and oyster samples were taken, respectively, and each group contained 3–5 individuals, which were used for testing of blocking efficacy. Six additional groups of shrimp and oyster samples were used for further method validation. All samples were obtained during the same period from the same pond.

Total DNA was extracted from the gut contents of the shrimp and oyster samples using the Hipure Mollusc DNA Kit (D3128-03, Magen, China). The Hipure Water DNA Kit (D3145-02, Magen, China) was used to extract DNA of the organisms (eukaryotic microorganisms and zooplankton). The 500 ml water sample collected from the pond was initially filtered through a filter screen (75 μm) (1werw45, Tengshuoge, China), and then filtered through filter membranes (0.22 μm) (C180096, PALL, United States) to obtain eukaryotic microorganisms in the water; Zooplankton was collected from around 15 L pond water using a plankton collector (Purity, China) (the mesh size of 0.16 mm). And then the condensed 100 mL collections were filtered onto the filter membranes (0.22 μm) to fix and preserve zooplankton. The DNA integrity was checked on a 1.5% agarose gel, and the DNA concentration was measured using a NanoDrop2000 spectrophotometer (Thermo Fisher Scientific, United States). Finally, the extracted DNA was stored at −20°C.

### Amplification With Blocking Primers

All the sample DNAs from the *L. vannamei*, *C. hongkongensis*, organisms in pond water were amplified in duplicate PCRs at a 25 μL reaction volume, containing 10 μL Platinum^TM^ Hot Start PCR Master Mix (2X) (13000014, Thermo Fisher Scientific, Lithuania), 1 μL (each) primer 1391f and primer EukBr (10 μM), 1 μL template DNA, 8–12 μL nuclease-free water, and 0–4 μL blocking primer (10 μM). The PCR thermal cycling conditions were as follows: 3 min at 94°C; 35 cycles of 30 s at 94°C, 30 s at 55°C, 1 min at 72°C; and finally 10 min at 72°C. The PCR products were checked by electrophoresis on a 1.5% agarose gel stained with DNA_GREEN_ (UV) (TIANDZ). In the electrophoresis results, the decrease in intensity of the band compares to the no-blocking, reflects the inhibitory effect of the blocking primer on the host DNA. The less intense the band, the better the blocking effect.

### Library Construction and Sequencing

#### Library Construction From PCR-Amplified Products

Following the results of the previous experiment, we could evaluate the inhibitory effects of the 10 blocking primers on the host sequences. 6 samples of *L. vannamei* and *C. hongkongensis* were amplified without and with the blocking primer, X-BP2-DPO at the concentrations of 0.4 and 0.6 μM in PCRs. The amplification of each sample was repeated thrice using PCR. Indexing PCRs were done using the following recipe: 1 μL template DNA, 1 μL forward primer with barcode (10 μM), 1 μL reverse primer with barcode (10 μM), 10 μL PCR master mix (2X), a range of volumes of the blocking primer X-BP2-DPO (0, 1, and 1.5 μL, the concentrations of 0, 0.4, and 0.6 μM in the PCR mixture), nuclease-free water up to 25 μL. Indexing PCRs were carried out on a TaKaRa PCR Thermal Cycler Dice (TP600) using the following cycling conditions: 94°C for 3 min and 35 cycles of: 94°C for 30 s, 55°C for 30 s, 72°C for 1 min, and 72°C for 10 min.

#### Library Normalization, Pooling, Quantification, and Sequencing

For the construction of the library, we normalized the samples by quantifying the indexing PCR products (three replicates from each sample were pooled) using a Qubit^^®^^ 3.0 Fluorometer (Invitrogen, Malaysia). We then normalized them to an equal concentration, pooled each normalized sample, purified and concentrated them with a 1X AMPure XP (Beckman Coulter) clean up kit, and finally eluted them in 50 μL Qiagen buffer (TE) for a library. The libraries were generated using PCR with Illunina adapters connected (NEB, United States), then were sequenced using an Illumina Hiseq 2500 System by Novogene company (Beijing, China). High-throughput sequence data are in NCBI’s Sequence Read Archive under SRA accession: SRP151638^3^.

#### Sequence Analysis

Sequencing data of the library were primarily analyzed using QIIME2^4^. The library containing sequences of several samples was demultiplexed based on the sequencing barcodes. We performed filtration and quality control of the sequences by applying the “DADA2” method (Callahan et al., 2016), which is used to infer sample sequences exactly and resolve differences of as little as 1 nucleotide^5^. The “DADA2” quality control process additionally filtered the phix reads and chimeric sequences, if any. The changes in the number of reads after filtration and quality control (Supplementary Table S3), and the quality of the library sequences (Supplementary Figure S2) are shown in Supporting Information. The feature table (i.e., abundance table of sequence variants per sample) (Bokulich et al., 2018) and representative sequence were generated by QIIME2 (data sheet.zip in Supporting Information), and the reads were identified using the qiime2 feature-classifier method using the SSU rRNA gene SILVA128 database^6^ (Quast et al., 2013) as reference.

#### Potential Inhibition Efficacy of X-BP2-DPO to Other Taxa

In order to estimate the potential inhibition efficacy of X-BP2-DPO to other taxa beyond this study, the primer sequence was further tested against the SILVA database (Release 132), using Diamond (version 0.9) (Buchfink et al., 2015), with the e-value set at 1e-5 for the alignment. All hits with alignments were ranked by their hit score and grouped based on their taxonomy origin (Figure 8).

## Results

### Selection of Blocking Primer

**FIGURE 1:**
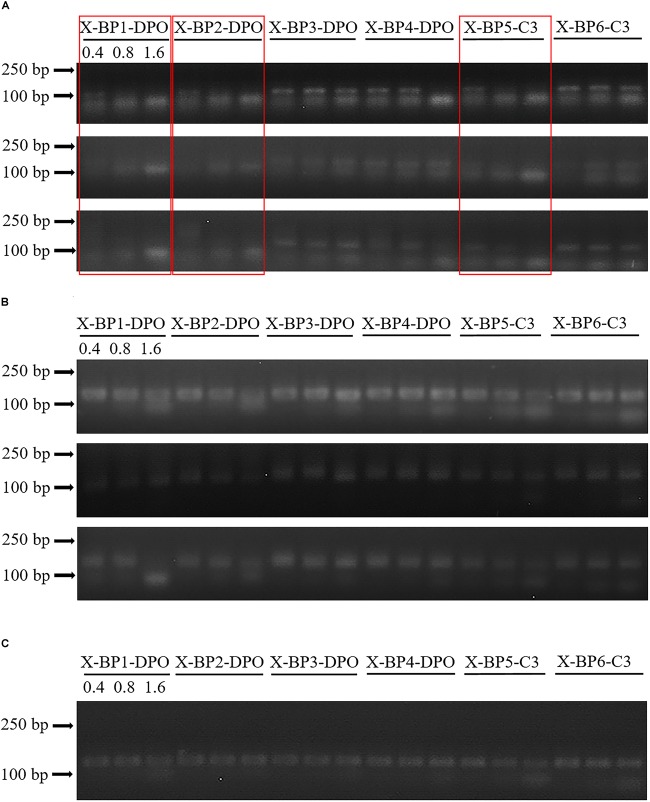
Inhibitory effects of blocking primers at varying concentrations against shrimp sequence amplification **(A)**, eukaryotic microorganisms **(B)**, zooplankton **(C)** templates. Samples were amplified with six different blocking primers against three shrimps at concentrations of 0.4, 0.8, and 1.6 μM. Experiments were repeated thrice. Primers (lanes) with preferred inhibition efficacy are marked with red rectangles. Please find the detailed methods of sample collection and PCR protocol from Sections “Sample Collection and DNA Extraction” and “Amplification With Blocking Primers” in the main text.

According to the brightness of the target bands in the electrophoresis, we could recognize the appearance of the inhibitory effects on host sequences (Figure 1A and Supplementary Figure S4). The fragment >100 is the target fragment (18S V9), and <100 are primer dimers. Quantification of the PCR bands by Quantity One (Version 4.6.6) allowed an easy detection of the inhibition. Primer, X-BP1-DPO, X-BP2-DPO, and X-BP5-C3 exhibited relatively good inhibitory effects on the amplification of both shrimp (Supplementary Figure S3A) and oyster (Supplementary Figure S3D) templates. The inhibitory effect on the host DNA with the blocking primers at the concentration of 0.8 or 1.6 μM is generally better than that at the concentration of 0.4 μM.

It was reported that species-specific blocking primers may block other non-host sequences as well (Boessenkool et al., 2012; Shehzad et al., 2012). Considering the fact that the blocking primers have a strong inhibitory effect on shrimps (Figure 1A and Supplementary Figure S3A) and a relatively good inhibition on oysters (Supplementary Figures S3D, S4), we were concerned about the inhibition of the primers at different concentrations on the non-host taxa. As shown in Figure 1B,C and Supplementary Figures S3B,C, we found the six primers against shrimp could hardly inhibit the amplification of the organisms at low concentrations (0.4 and 0.8 μM).

The inhibitory effects of the four blocking primers against oyster, L-BP1-C3, L-BP2-C3, L-BP3-C3, and L-BP4-C3 (Supplementary Table S2) were also evaluated. However, we did not find pronounced inhibitory effects of the primers on oyster DNA (Supplementary Figures S3E, S5). It was clear that the blocking primers against shrimp had better inhibition of oyster DNA than the blocking primers against oyster (Supplementary Figures S3D, S4). So, we continued to study the eukaryotic microbes in shrimps and oysters with the blocking primers against shrimp.

The performance of the blocking primer with non-host DNA was sensitive to its concentration in the PCR mixture; hence, keeping the blocking primer concentration at the minimum required amount is a good approach (Vestheim and Jarman, 2008). We then carried out an in-depth data analysis by high-throughput sequencing of the shrimp and oyster samples amplified with the blocking primer, X-BP2-DPO, which was chosen as the most effective primer based on its inhibition at the concentrations of 0, 0.4, and 0.6 μM in barcode PCRs followed by Illumina sequencing.

### In-Depth Data Analysis

#### Composition of Identifiable Taxa

Up to 4,035,147 reads for the library containing 18 samples were generated for 18S rDNA V9 amplicons from Hiseq2500, with an average of 144,723 reads per sample after quality filtering. As the readable length of Hiseq2500 was adequately long for the 18S V9 amplicon, we did not merge paired-end reads, we simply used read1 for subsequent analysis.

Supplementary Figure S6 shows the observed feature (ASV, amplicon sequence variants) numbers of the different samples at increasing sequencing depths. A sequencing depth of 20000 seemed to be largely sufficient despite the treatment and 12000–20000 appears to be an appropriate depth range when performing high-throughput sequencing of the 18S rRNA genes in shrimp and osyster gut. The gut contents of marine animals always include a large diversity of marine organisms, especially low-abundant taxa (Osei-Poku et al., 2012; Su et al., 2017). The use of blocking primers seemed to increase the overall numbers of ASVs per sample. All reads from all samples were clustered into 1,255 ASVs after sequencing and analyzing. The taxonomy distribution of the representative sequences determined by sequence similarity against the Silva database version 128 allowed the identification of 105 distinct taxa. Taxonomic composition and ASV table of all the samples is shown in Supplementary Tables S4, S5.

**FIGURE 2:**
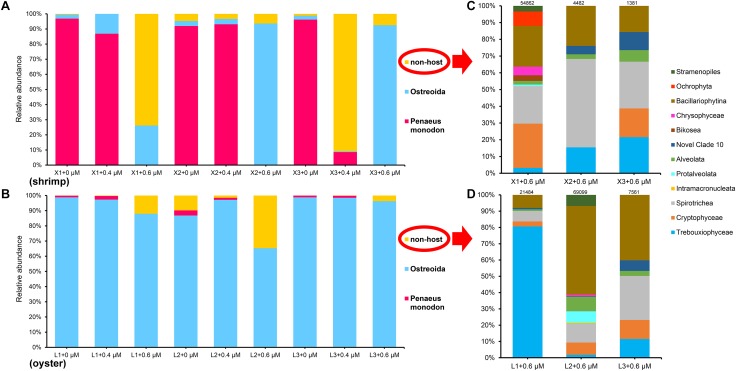
The taxonomic composition of eukaryotic microorganisms in shrimp **(A,C)** and oyster **(B,D)** amplified with the blocking primer at various concentrations. **C** and **D**, details of class composition of “non-host” in shrimp and oyster samples (the relative abundance of ASVs >0.01%) when the primer concentration is 0.6 μM. Numbers above each bar chart are reads counts, and were all grouped in class level. “+0,” “+0.4,” and “+0.6 μM” implying that the samples were amplified with the blocking primer, X-BP2-DPO, at different concentrations. “X1-3” and “L1-3” are six different shrimp and oyster samples, respectively.

In Figure 2A, it is obvious that the dominant host DNA (about 93%) and very few eukaryotic microorganisms are present in the shrimp samples amplified without any blocking primes; the host DNA content decreased markedly with the addition of the blocking primer, X-BP2-DPO, at a concentration of 0.4 μM during PCR; and then, the host DNA content became minor with a large diversity of eukaryotic microorganisms in shrimp samples upon the addition of a blocking primer at 0.6 μM (Figure 2C). Sequencing results demonstrated the primer’s ability to amplify diverse eukaryotic microorganisms while the host DNA amplification was inhibited to represent a total 0.01% of the total reads when using the blocking primer at 0.6 μM in shrimp samples.

Figure 2D also shows a relatively rich microbial diversity in oyster samples with the primer concentration at 0.6 μM, but the primer’s inhibitory effects were not as effective on oyster DNA as was for shrimp (Figure 2B, Supplementary Figure S7).

#### Proportion of Taxa

Among the 105 annotated taxa in the shrimp and oyster samples, the dominant identifiable taxa include hosts, *Cyclotella*, Cryptomonadales, *uncultured Halteria*, and Oligotrichia.

**FIGURE 3:**
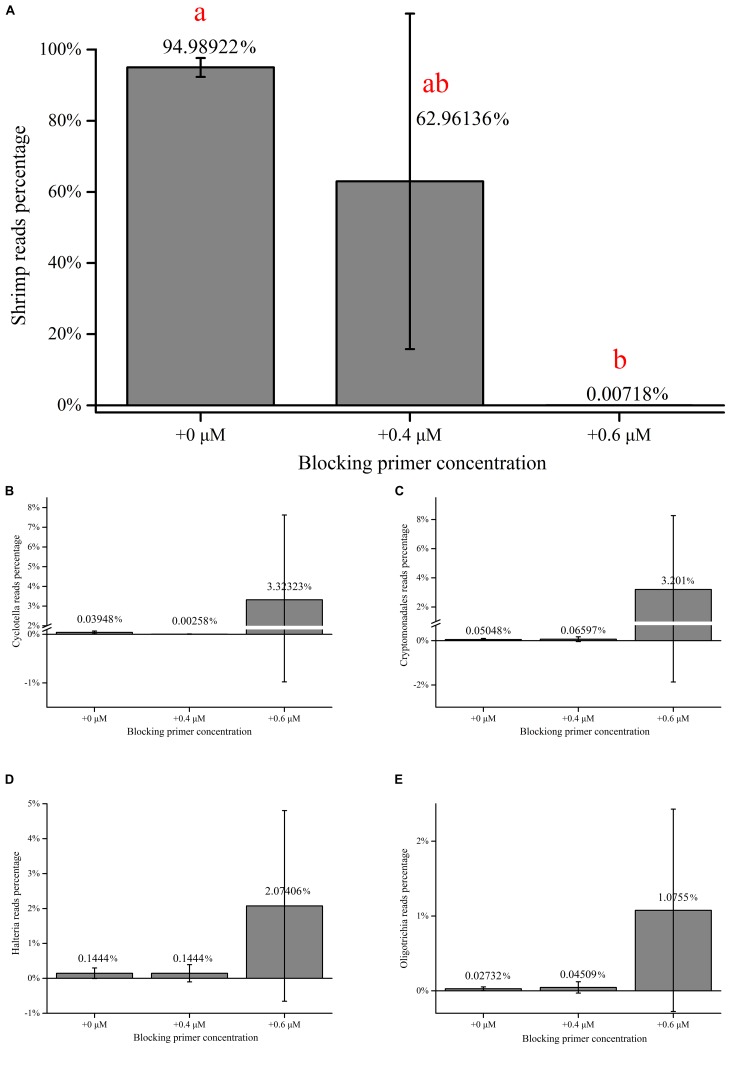
Changes in the percentage of taxa in shrimp samples. **(A)** Changes in the percentage of host reads in shrimp samples amplified with the blocking primer, X-BP2-DPO at various concentrations. **(B–E)** Changes in percentage of the four dominant eukaryotes reads [*Cyclotella* **(B)**, Cryptomonadales **(C)**, *uncultured Halteria* **(D)**, and Oligotrichia **(E)**] in shrimp samples amplified with the blocking primer, X-BP2-DPO at various concentrations. “+0,” “+0.4,” and “+0.6 μM” imply that the samples were amplified with the blocking primer, X-BP2-DPO at the concentrations of 0, 0.4, and 0.6 μM in the PCRs. The data was analyzed by applying Tukey’s test and Levene’s test (*p* = 0.013).

For shrimp samples, there is a significant difference between the proportions of shrimp host when the primer concentration is 0 and 0.6 μM (Figure 3A). The proportions of the four dominant eukaryotes increased clearly, while the host reads were almost completely suppressed with an addition of the primer at 0.6 μM (Figure 3). A quite rich eukaryotic microflora was detected by the 18S universal primers with the addition of the blocking primer at 0.6 μM simultaneously, which explains the effectiveness of the blocking primer for the shrimp samples.

**FIGURE 4:**
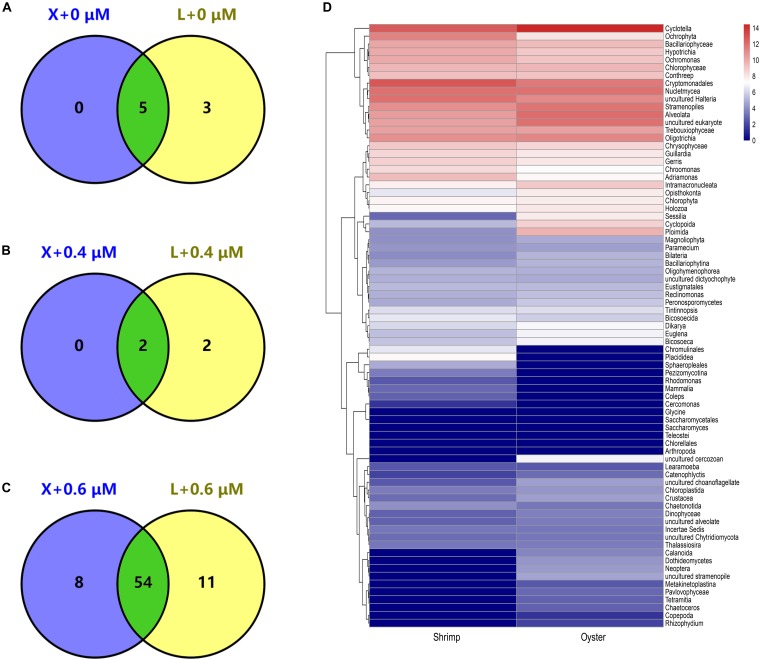
Detected genus of eukaryotic microorganisms shared between shrimp and oyster samples. “X” and “L” represent shrimp and oyster samples, respectively. They were amplified with X-BP2-DPO at different concentrations (0, 0.4, and 0.6 μM). **A–C** are Venn diagrams; the number in the circle represents the number of genera detected in shrimp and/or oyster samples. **D** is a heatmap of the overall composition of the eukaryotic microorganisms amplified with X-BP2-DPO at 0.6 μM [select log2(X + 1) for data visualization, from blue to red, the number is gradually increasing.].

Venn diagrams show the diversity of taxa present in shrimp and oyster hosts amplified with the blocking primer at different concentrations. The number of common taxa present in the two kinds of samples increased significantly with the addition of the blocking primer in PCRs (Figure 4A,C). The addition of primers increases the proportion of eukaryotic microorganisms, which is likely to be related to the diet of shrimps and oysters. In Figure 4D shows 54 common eukaryotic microorganisms detected both in shrimp and oyster guts, including a large variety of algae, protists, eumycetes. The percentage of most eukaryotic microorganisms (algae, protists) is less than 1% of total taxa population. The top few taxa that are well-annotated include *Cyclotella*, Cryptomonadales, Ochrophyta, Bacillariophyceae, Trebouxiophyceae, *uncultured Halteria*, Oligotrichia, Stramenopiles, Alveolata, and Nucletmycea.

#### Diversity Index Analysis

As expected and shown in Tables 1, 2, the changes in these indices (“observed_otus,” “ace,” “Chao1,” “shannon,” “faith_pd,” “pielou_e”) are almost positively related to the richness of identifiable taxa in shrimp and oyster samples. The richness and evenness of shrimp and oyster samples amplified with the blocking primer were higher than those amplified without the primer. In general, the optimal (maximum) value of each index results while the primer concentration used is 0.6 μM.

**Table 1:** Diversity index of reads in shrimp samples.

Diversity index	X1 + 0^a,b1^	X1 + 0.6	X2 + 0	X2 + 0.4	X2 + 0.6	X3 + 0	X3 + 0.4	X3 + 0.6
Filtered reads	40039	74312	39202	18912	69906	63523	799673	18395
Observed_otus^c^	16	323	35	34	112	40	49	75
Ace^d^	16.2196	335.802	35	34	117.459	42.1564	62.7226	75
Chao1^e^	16	332.45	35	34	115.714	40.6	61	75
Simpson^f^	0.13019	0.92386	0.2204	0.569	0.24483	0.16207	0.74775	0.2602
Shannon^g^	0.49718	5.43345	0.91121	1.58459	1.07654	0.63989	2.78415	1.18267
Faith_pd^h^	5.43251	19.2559	6.42457	7.11525	9.10699	6.84896	5.18038	9.12366
Pielou_e^i^	0.1243	0.65185	0.17765	0.31147	0.15814	0.12024	0.49587	0.18987

**Table 2:** Diversity index of reads in oyster samples.

Diversity index	L1 + 0^a,b2^	L1 + 0.4	L1 + 0.6	L2 + 0	L2 + 0.4	L2 + 0.6	L3 + 0	L3 + 0.4	L3 + 0.6
Filtered reads	37890	19229	178091	140748	16653	199258	153608	471173	200992
Observed_otus^c^	14	9	179	34	15	302	16	30	144
Ace^d^	14.249	9.5175	217.984	38.7974	15	340.907	18.1536	38.0866	196.072
Chao1^e^	14	9	216.5	39.25	15	337.935	17	35	183
Simpson^f^	0.24506	0.24456	0.3265	0.40477	0.24681	0.62987	0.22252	0.34249	0.19738
Shannon^g^	0.67535	0.72981	1.36916	1.52014	0.76867	3.18205	0.63619	0.86991	0.82696
Faith_pd^h^	4.29526	5.84358	13.6546	4.26958	4.12717	23.8013	3.83661	3.27697	10.9164
Pielou_e^i^	0.17738	0.23023	0.18295	0.2988	0.19675	0.38625	0.15905	0.17728	0.11534

*^a^“X + 0,” “X + 0.4,” and “X + 0.6” mean that the shrimp samples were amplified with the blocking primer, X-BP2-DPO at the concentrations of 0, 0.4, and 0.6 μM. ^b1^“X1, X2, and X3” are three different shrimp samples. ^b2^“L1, L2, and L3” are three different oyster samples. ^c^“observed_otus” index is studied to calculate numbers of distinct OTUs (DeSantis et al., 2006). ^d^Abundance-based Coverage Estimator (ace) metric and ^f^“Simpson” index are used to estimate species richness using a correction factor (Simpson, 1949; Chao and Lee, 1992). ^e^“Chao1” index and ^g^“Shannon” index indicate the diversity from abundant data (Shannon et al., 1951; Chao, 1984). ^h^Faith’s phylogenetic diversity (faith_pd) index is the measures of biodiversity that incorporates phylogenetic difference between species (Faith, 1992). ^i^Pielou’s evenness (pielou_e) index measures the relative evenness of species richness (Pielou, 1966), which is proportional to changes in species richness. Sample “X1 + 0.4” was filtered out when performing rarefaction of samples because of its very low number of reads (the sampling depth was 16653).*

#### Comparative Analysis

**FIGURE 5:**
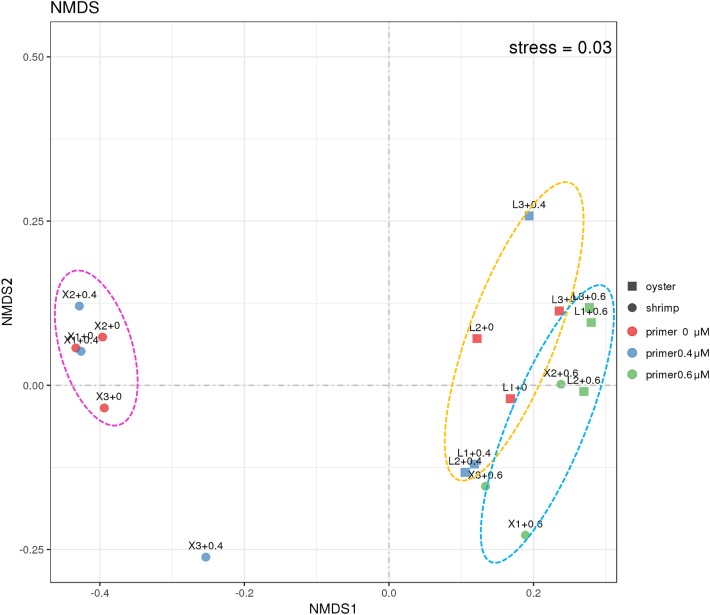
NMDS analysis of the 18S community in shrimp and oyster samples. Stress is the measure of the degree of dissimilarity between the object structure and the original distance matrix in the sort space based on Kruskal stress formula I. When stress <0.05, it means that the sample is highly representative based on Bray-Curtis distance between the 18S ASVs composition of different samples (Netburn and Koslow, 2018). “+0,” “+0.4,” and “+0.6” imply shrimp (circles) and oyster samples (square) were amplified with the blocking primer, X-BP2-DPO at the concentrations of 0 μM (red), 0.4 μM (blue), and 0.6 μM (Green) in PCRs.

In order to learn more about the differences between the samples amplified with and without the blocking primer, NMDS (non-metric multi-dimensional scaling) analysis was used (Figure 5). It is calculated based on the Bray-Curtis distance between samples (R), reflecting the nonlinear structure of the sequence data (Kenkel and Orlóci, 1986; Thibodeau et al., 2015). From the result, it was observed that the shrimp and oyster samples represent good aggregations, respectively, and the samples amplified with blocking primer at 0.6 μM in PCRs tend to cluster together. The samples show a good representation according to the stress (<0.05), which indirectly validates the reliability of the sample sequences.

**FIGURE 6:**
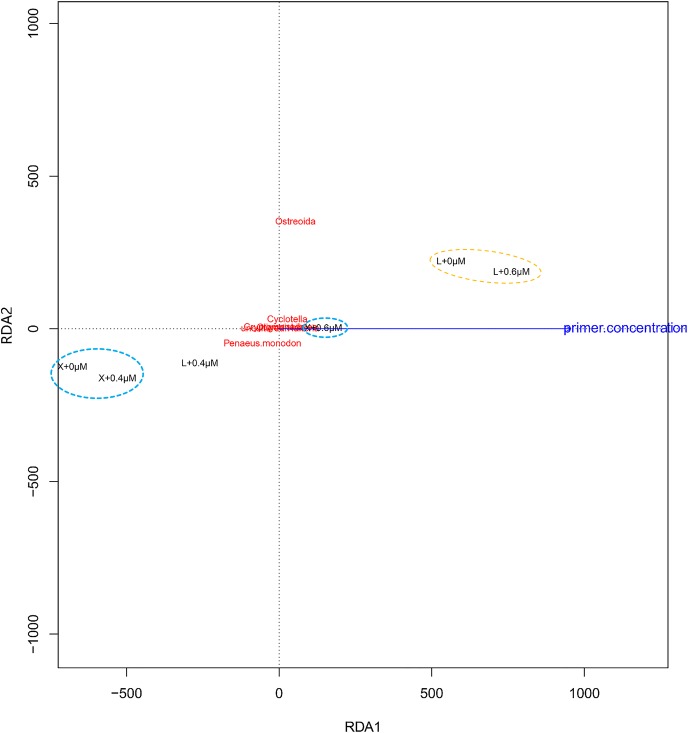
Redundancy analysis (RDA) of the samples and the blocking primer. “+0,” “+0.4,” and “+0.6 μM” imply that the samples were amplified with the blocking primer, X-BP2-DPO at the concentrations of 0, 0.4, and 0.6 μM in the PCRs. “X” and “L” represent the shrimp and oyster samples, respectively.

Redundancy analysis (RDA) is a method to extract and summarize the variation in a set of response variables that can be explained by a set of explanatory variables (van den Wollenberg, 1977). Figure 6 (RDA) showed the shrimp samples “X + 0.4 μM” and “X + 0 μM,” the oyster samples “L + 0 μM” and “L + 0.6 μM” aggregate separately, and the taxa of high proportion in samples maintain good aggregation as well. Compared to the Ostreoida (*C. hongkongensis*), shrimp (*P. monodon*) seems to be more closely associated to a few main eukaryotes (*Cyclotella*, *Cryptomonadales*, *uncultured Halteria*, *Oligotrichia*).

#### Further Verification

**FIGURE 7:**
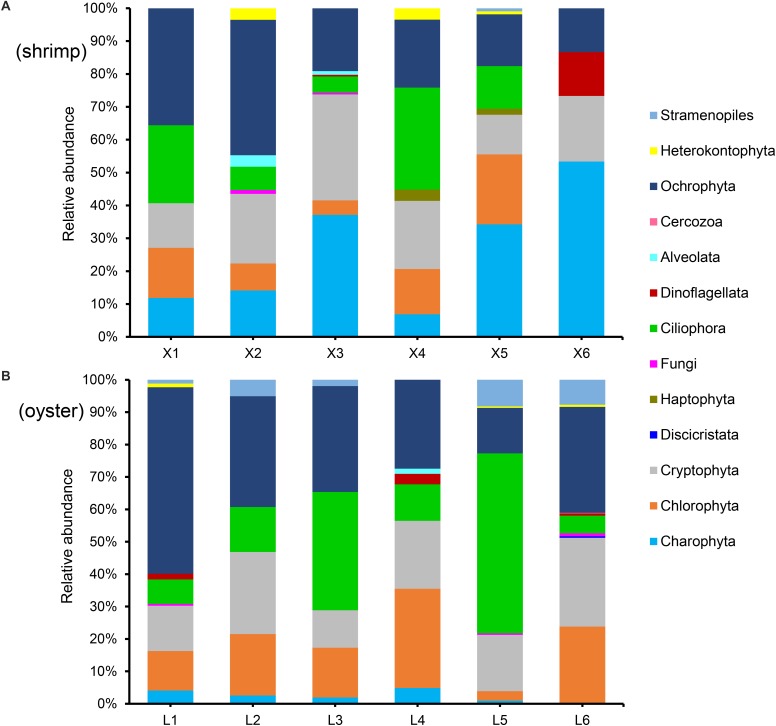
Phylum composition (>0.01%) of shrimp **(A)** and oyster samples **(B)**, amplified with the blocking primer, X-BP2-DPO, at 0.6 μM PCR concentration. X1-6 and L1-6 are six different shrimp and oyster samples, respectively.

In order to further verify the effect of blocking primer, we evaluated X-BP2-DPO with another six repetitions of shrimp (Figure 7A) and oyster (Figure 7B) at the concentration of 0.6 μM. The result showed that abundant eukaryotic microorganisms at phylum level were detected out from both shrimps and oysters and their composition varied significantly among different individuals.

**FIGURE 8:**
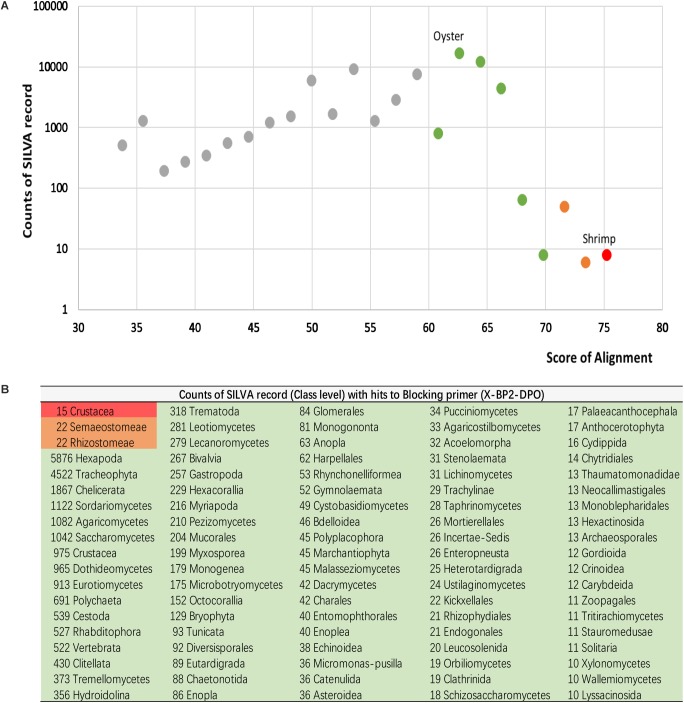
Alignments for primer sequences and the SILVA database (Release 132). All hits with alignments were ranked by their hit score **(A)**, and grouped based on their taxonomy origin **(B)**.

However, as only a few sequences were included in the primer design, it is uncertain whether the blocking primer could be incidentally biased against another taxonomic group beyond this study. Therefore, the sequence of X-BP2-DPO was further tested against the SILVA database. As the primer sequence was based on the 18S universal primer (1391f), it was aligned mostly at the 5 end 89.8% (69604/77541) to SILVA records. The sequences with ambiguous taxonomy were excluded, and the remaining 51833 sequences were grouped into four major groups based on their hit score. As shown in Figure 8, shrimp sequences (15 records) were assembled in the first group, the group with the highest hit score (Figure 8A,B, red highlight) and with alignments not only at the 5′ but also at the 3′ end, very important to PCR efficacy. Meanwhile, 44 jellyfish records (Semaeostomeae and Rhizostomeae) were also blasted with high score (>70, orange highlight). However, these hits did not have alignments at the 3′ end. Thus, the efficacy in blocking the amplification would be probably lower in jellyfish than in shrimp. Bivalves (267 records), together with abundant Hexapoda, Tracheophyta, and Chelicerata and other records, were assembled in the third group with hit scores between 60 and 70 (green highlight). Records with lower score (<60) were assembled in the fourth group, the group with the least inhibition efficacy.

## Discussion

Although the bacterial component of the microbiome has been the focus of most microbial diversity research to date, viruses, prokaryotes, and eukaryotic microbes together form a complex network of ecological interactions that ultimately defines the complete microbiome (Belda et al., 2017). A blocking primer designed to suppress PCR amplification of metazoan DNA in protistan community by Tan and Liu (2018) can reduce 42.1–72.4% of metazoan sequences; Belda et al. (2017) has developed Anneal-inhibiting blocking primers and PNA oligonucleotide blockers to reduce the amplification of mosquito 18S rRNA gene sequences by 80% when studying the mosquito eukaryotic microbiome; To select the amplification of bacterial DNA from microalgae cultures, Powell et al. (2012) developed a blocking primer to reduce the proportion of algal chloroplasts sequences was in clone library from 70% to only 6%. In this study, the 99% inhibition rate of the blocking primer, X-BP2-DPO against shrimp host sequences outperformed above studies.

We focused on improving the simplicity and efficiency of strategy for blocking the eukaryotic host DNA amplification and detecting eukaryotic microorganism diversity in the gut of marine animals. The addition of a blocking primer is a simple method that allows for using a single PCR to simultaneously and distinctly amplify the eukaryotic microorganisms amongst a vast quantity of host DNA in each sample. The effect of the blocking primer on host seems better when 44444 and C3 are added together at the 3′-end (Figure 1A). The blocking primer was intended to selectively bind to the host sequence and ended with five deoxyinosine molecules and a C3 spacer that prevent extension during the PCR of the host target without noticeably influencing the annealing properties of primer. At the same time, other eukaryotes (such as protist, algae, fungi, etc.) in the samples were abundantly amplified by the 18S universal primer. Applications of the blocking primers against marine animals help to understand the composition and characteristics of eukaryotic microbes in the guts in order to make a further study on their diets. The relevant primers with some modifications could even promote the research of the detection of parasitic pathogens in animals.

Considering the blocking primer against shrimp, X-BP2-DPO may also mildly inhibit the eukaryotic microorganisms in hosts (Boessenkool et al., 2012; Shehzad et al., 2012), keeping the blocking primer concentration at the minimum required amount is a matter of considerable interest, though such inhibition is not evident from the Figure 1B,C and Supplementary Figures S3B,C). Surprisingly, the blocking primer against shrimp has a better inhibition on the amplification of oyster DNA than the primer against oyster from Supplementary Figures S3D,E, S4, S5. So, we analyzed the eukaryotic microbes in shrimps and oysters using the blocking primers against shrimp, X-BP2-DPO.

However, there seems to be some inconsistencies in our results, for example: The annotation information obtained in silva_128_database for the proportions in Sample, X3 + 0.4 μM through the present annotation process by QIIME2 could only be accurate to “Eukaryota” (Supplementary Table S4), which may be associated with the incompleteness of SILVA database or that the sequenced region does not allow unambiguous identification. We found the large proportion annotated as “Eukaryota” is mainly composed of *Cryptomonas* sp. 18S rRNA gene, *Crassostrea gigas* 18S rRNA gene, and *Goniomonas avonlea* 18S rRNA gene using the NCBI blast; it is not hard to notice that a large part of oyster DNA (Ostreoida) exists in shrimp samples, especially in Sample “X2 + 0.6 μM” and “X3 + 0.6 μM” (Figure 2A). A possible cross contamination of PCRs can be ruled out since we had set negative controls which show no amplification, and the amplification of positive samples with variable microbial biodiversity means that the apparent “contamination” was not from the laboratory. In the ecosystem of shrimp and shellfish polyculture farming, shrimps and oysters are the two most important species at a high nutritional level (Martinez-Cordova and Martinez-Porchas, 2006). *L. vannamei* is an omnivorous scavenger (Hill and Wassenberg, 1987), and *C. hongkongensis* could filter large amounts of water (Shpigel and Blaylock, 1991; Liudong et al., 2017), which would have an impact on shrimps. In fact, oyster DNA is present in shrimp samples, and shrimp DNA is also present in oyster samples in the results of the compositional analysis (Figure 2). There are a lot of free DNA in the pond water, it is likely that some oyster DNA ends up in the shrimp diet and vice versa.

To study the diet of shrimps (*L. vannamei*) and oysters (*C. hongkongensis*), we examined the compositional characteristics of the common species present in the shrimp and oyster hosts. Predator-prey interactions could ultimately determine the fate and flux of every trophic level in an ecosystem, particularly the upper-level consumers of economic importance (Link, 2004). Considering the omnivorous nature of shrimps and the filter-feeding properties of oysters, large numbers of eukaryotic microorganisms in aquaculture water are likely to enter shrimps and oysters during feeding, resulting in similarities in their diet. Among the 67 common species, the top high-content taxa include *Cyclotella*, Cryptomonadales, Ochrophyta, Bacillariophyceae, Trebouxiophyceae, *uncultured Halteria*, Oligotrichia, Stramenopiles, Alveolata, Nucletmycea, etc. As the major consumers in the ecosystems of shrimp and shellfish polyculture farming, shrimps and oysters have contributed to the web structure of the microbial community. The above-mentioned eukaryotic microorganisms (protist, eukaryotic algae, and fungi) may are derived from aquaculture water, and some fungi may also come from certain culture food supplements (yeast, probiotics, etc.) (Burgents et al., 2004). The use of our blocking primer allowed detection of small amounts of eukaryotic microorganisms.

## Conclusion

Predator–prey interactions in marine ecosystems are of critical importance in the structuring of marine communities and determining the health of the world’s oceans (Bailey et al., 2010). The host-specific blocking primer against *L. vannamei*, X-BP2-DPO is a highly promising tool to inhibit the 18S rDNA amplification of this shrimp species. Addition of the blocking primer could greatly enhance the quantity and the diversity of the eukaryotic microorganisms in shrimps, thereby improving our ability to maximize the gut content information about them in favor of studying the taxa composition and their relationships with environment. The development of blocking primers will offer more universal applicability to different marine animals based on the guarantee of simplicity and efficiency of the blocking primer against specific species.

## Ethics Statement

All animal work have been conducted according to relevant national and international guidelines. South China Sea Fisheries Research Institute Academic Committee approved this research.

## Author Contributions

CL, R-JQ, and J-ZJ conceived and planned the experiments. CL, R-JQ, and M-QZ carried out the experiments. CL analyzed the sequencing data. J-ZJ and J-YW helped to supervise the experiments and analysis. CL and J-ZJ wrote the manuscript. All the authors provided critical feedback and helped to shape the research, analysis and manuscript.

## Conflict of Interest Statement

The authors declare that the research was conducted in the absence of any commercial or financial relationships that could be construed as a potential conflict of interest.

## Footnotes

1 https://www.ncbi.nlm.nih.gov

2 http://www.earthmicrobiome.org/protocols-and-standards/18S/

3 https://www.ncbi.nlm.nih.gov/sra/SRP151638

4 https://qiime2.org/

5 https://github.com/benjjneb/dada2/

6 https://www.arb-silva.de/download/archive/qiime/Silva_128_release.tgz
