# Supplementary material for: Development of a Blocking Primer to Inhibit the PCR Amplification of the 18S rDNA Sequences of Litopenaeus vannamei and Its Efficacy in Crassostrea hongkongensis
Source: Front Microbiol. 2019 Apr 23;10:830. doi: 10.3389/fmicb.2019.00830 (PMC6489566; doi:10.3389/fmicb.2019.00830)
Supplement: Supplementary file 2 [file Data_Sheet_2.zip › Supplementary Figures and Tables.doc]

Supporting information

# Development of a blocking primer to inhibit the PCR amplification of the 18S rDNA sequences of *Litopenaeus vannamei* and its efficacy in *Crassostrea hongkongensis*

Cong Liu ^1,2,†^, Ren-Jie Qi ^1,3,†^, Jing-Zhe Jiang ^1,*^, Ming-Qing Zhang^4^, Jiang-Yong Wang ^1,*^

Affiliations

1 Key Laboratory of Aquatic Product Processing, Ministry of Agriculture and Rural Affairs, South China Sea Fisheries Research Institute, Chinese Academy of Fishery Sciences, Guangzhou 510300, China

2 College of Fisheries and Life Science, Shanghai Ocean University, 200090, Shanghai, China

3 Tianjin Agricultural University, Tianjin 300384

4 Guangzhou University, Guangzhou 510006

† These authors contributed equally to the work.

* Corresponding Author

Jiang Jing-Zhe

E-mail: [jingzhejiang@gmail.com](mailto:jingzhejiang@gmail.com)

Telephone: 86-20-89022636

Wang Jiang-Yong

E-mail: [wjy104@163.com](mailto:wjy104@163.com)

Telephone: 86-20-89108321

Fig. S1A 5′ Alignment of algae, shrimp, and protozoan at 5′

Fig. S1A 3′ Alignment of algae, shrimp, and protozoan at 3′

Fig. S1B 5′ Alignment of shrimp and protozoan at 5′

Fig. S1B 3′ Alignment of shrimp and protozoan at 3′

Fig. S1C 5′ Alignment of oyster and algae at 5′

Fig. S1C 3′ Alignment of oyster and algae at 3′

Fig. S1D 5′ Alignment of oyster and protozoan at 5′

Fig. S1D 3′ Alignment of oyster and protozoan at 3′

Fig. S1E 5′ Alignment of oyster, protozoan, and algae at 5′

Fig. S1E 3′ Alignment of oyster, protozoan, and algae at 3′

Fig. S1 Alignment of species. Fig. S1A–S1E shows the exact match between the 18S rDNA sequences of the various eukaryotes such as shrimp, oyster, eukaryotic algae, and protist by intercepting the same fragment of their sequences. All alignments include the 18S rDNA sequences of several eukaryotes, which were obtained from GenBank® ([http://www.ncbi.nlm.nih.gov](http://www.ncbi.nlm.nih.gov/)). ‘5′’ indicates the sequences at the 5′ end, and ‘3′’ indicates the sequences at the 3′ end.

Fig. S2 Quality of the library sequences

Fig. S3 Inhibition efficacy of different 18S rDNA blocking primers at various concentrations. The X axes show different blocking primers, and the Y axes show the percentages of band brightness remaining after adding blocking primers. Different templates, *Litopenaeus vannamei* (A), eukaryotic microorganisms (B), zooplankton (C), and *Crassostrea hongkongensis* (D and E), are evaluated. A–C show the results of 6 blocking primers (X-BP*) designed against *L. vannamei* sequences at different concentrations (0.4, 0.8, and 1.6 μM). D shows the results of 3 blocking primers (X-BP*) tested using *C. hongkongensis* templates. E shows the results of 4 blocking primers (L-BP*) designed against *C. hongkongensis* sequences at different concentrations. A-1–3, B-1–3, and D-1–3 show three sample repetitions.

Fig. S4 Inhibitory effects of the blocking primers against shrimp on oyster DNA amplification at various concentrations. Bands between 100 and 250 bp are target products. Of the six blocking primers against shrimp tested, we found that the primers, X-BP1-DPO, X-BP2-DPO, and X-BP5-C3 could also inhibit the amplification of oyster DNA. The inhibitory effects on the oyster host with the blocking primers at 0.8 μM or 1.6 μM are better than that at 0.4 μM.

Fig. S5 Inhibitory effects of the blocking primers against oyster on oyster DNA amplification at various concentrations. Oyster samples amplified with the four types of blocking primers at the concentrations of 0.4 μM, 0.8 μM, and 1.6 μM in the PCRs were examined.

Fig. S6 Alpha rarefaction plot of the sequencing samples (A, shrimp samples; B, oyster samples). As the depth of sequencing increases, the ASVs number of the samples contained in the library increases and tends to be unchanging gradually. ‘+0 μM’, ‘+0.4 μM’, and ‘+0.6 μM’ imply that the samples were amplified with the blocking primer, X-BP2-DPO at the concentrations of 0 μM, 0.4 μM, and 0.6 μM in the PCRs. ‘X1, X2, X3’ and ‘L1, L2, L3’ are three different shrimp and oyster samples, respectively.

Fig. S7 Changes in the percentage of species in oyster samples.

Fig. S7A Changes in percentage of host reads in oyster samples amplified with the blocking primer, X-BP2-DPO at various concentrations. The data were analyzed by applying Tukey’s test and Levene’s test.

Fig. S7B-S7E Changes in percentage of the four dominant eukaryotes reads (*Cyclotella* (B), Cryptomonadales (C), *uncultured* *Halteria* (D), and Oligotrichia (E)) in oyster samples amplified with the blocking primer, X-BP2-DPO at various concentrations. ‘+0 μM’, ‘+0.4 μM’, and ‘+0.6 μM’ imply that the samples were amplified with the blocking primer, X-BP2-DPO at the concentrations of 0 μM, 0.4 μM, and 0.6 μM in the PCRs.

Table S1 List of the accession numbers of the 18S rDNA sequences of eukaryotic species (with annotation at Phylum level)

| **Group** | **Accession Number** | **Phylum (Species)** |
| --- | --- | --- |
| A | FR865635.1 | Rhodophyta (*Polysiphonia stricta*) |
|  | DQ629010.1 | Rhodophyta (*Lithothamnion sp. BISH 689378*) |
|  | KX109776.1 | Chlorophyta (*Chlorella sp. WT1*) |
|  | FM876988.1 | Rhodophyta (*Porphyridium sp. ex foraminifera MH-2008*) |
|  | AF124597.1 | Arthropoda (*Litopenaeus vannamei*) |
|  | EU920969.1 | Arthropoda (*Litopenaeus vannamei*) |
|  | AJ238069.1 | Arthropoda (*Chirocephalus diaphanus*) |
|  | AB374225.1 | Platyhelminthes (*Diphyllobothrium nihonkaiense*) |
|  | AJ250699.1 | Endomyxa (*Marteilia refringens*) |
| B | AF124597.1 | Arthropoda (*Litopenaeus vannamei*) |
|  | EU920969.1 | Arthropoda (*Litopenaeus vannamei*) |
|  | AJ238069.1 | Arthropoda (*Chirocephalus diaphanus*) |
|  | AB374225.1 | Platyhelminthes (*Diphyllobothrium nihonkaiense*) |
|  | AJ250699.1 | Endomyxa (*Marteilia refringens*) |
| C | AB064942.1 | Mollusca (*Crassostrea gigas*) |
|  | U88709.1 | Mollusca (*Ostrea edulis*) |
|  | L49052.1 | Mollusca (*Ostrea edulis*) |
|  | AY144598.1 | Mollusca (*Crassostrea rivularis*) |
|  | FR865635.1 | Rhodophyta (*Polysiphonia stricta*) |
|  | KX109776.1 | Chlorophyta (*Chlorella sp. WT1*) |
|  | FM876988.1 | Rhodophyta (*Porphyridium sp. ex foraminifera MH-2008*) |
| D | AB064942.1 | Mollusca (*Crassostrea gigas*) |
|  | U88709.1 | Mollusca (*Ostrea edulis*) |
|  | L49052.1 | Mollusca (*Ostrea edulis*) |
|  | AY144598.1 | Mollusca (*Crassostrea rivularis*) |
|  | AJ238069.1 | Arthropoda (*Chirocephalus diaphanus*) |
|  | AJ250699.1 | Endomyxa (*Marteilia refringens*) |
|  | KT767113.1 | Platyhelminthes (*Bothriocephalus scorpii*) |
| E | AB064942.1 | Mollusca (*Crassostrea gigas*) |
|  | U88709.1 | Mollusca (*Ostrea edulis*) |
|  | L49052.1 | Mollusca (*Ostrea edulis*) |
|  | AY144598.1 | Mollusca (*Crassostrea rivularis*) |
|  | FR865635.1 | Rhodophyta (*Polysiphonia stricta*) |
|  | KX109776.1 | Chlorophyta (*Chlorella sp. WT1*) |
|  | FM876988.1 | Rhodophyta (*Porphyridium sp. ex foraminifera MH-2008*) |
|  | AJ238069.1 | Arthropoda (*Chirocephalus diaphanus*) |
|  | AJ250699.1 | Endomyxa (*Marteilia refringens*) |
|  | KT767113.1 | Platyhelminthes (*Bothriocephalus scorpii*) |

Table S2: Blocking primers

| Primer name | Sequence (5′→3′) |
| --- | --- |
| X-BP1-DPO | GTACACACCGCCCGTCGCTACTACCGATTGAATGGTCTAGTGA**44444**GGGCAC**3** |
| X-BP2-DPO | GTACACACCGCCCGTCGCTACTACCGATTGAATGGTCTA**44444**GTGAGGGCAC**3** |
| X-BP3-DPO | ACCGCCCGTCGCTACTACCGATTGAATGGTCTA**44444**GTGAGGGCAC**3** |
| X-BP4-DPO | ACCGCCCGTCGCTACTACCGATTGAATGGTCTAGTGA**44444**GGGCAC**3** |
| X-BP5-C3 | GTACACACCGCCCGTCGCTACTACCGATTGAATGGTCTAGTGAGGGCAC**3** |
| X-BP6-C3 | ACCGCCCGTCGCTACTACCGATTGAATGGTCTAGTGAGGGCAC**3** |
| L-BP1-C3 | GTACACACCGCCCGTCGCTACTACCGATTGGATGGTTTAGTGAGCYCCTT**3** |
| L-BP2-C3 | GTACACACCGCCCGTCGCTACTACCGATTGGATGGTTTAGTGAGCYCCTTGGACTGGTCC**3** |
| L-BP3-C3 | GTACACACCGCCCGTCGCTACTACCGATTGGATGGTTTAGTGAGCYCCTTGGACTGGTCCCGAC**3** |
| L-BP4-C3 | GTACACACCGCCCGTCGCTACTACCGATTGGATGGTTTAGTGAGCYCCTTGGACTGGTCCCGACGGCGCGG**3** |

4: deoxyinosine molecules

3: C3 spacer

X: *L. vannamei*; L: *C. hongkongensis*.

The primers marked with ‘X’ are designed against the shrimp host; the primers marked with ‘L’ are designed against the oyster host.

BP: blocking primer

DPO: a dual priming oligonucleotide (Chun et al., 2007).

Table S3 List of the changes in reads numbers in the library after filtration and quality control

| **Samples** | **Rawdata** | **Filtered and Quality-controlled** |
| --- | --- | --- |
| X1+0 μM | 47272 | 40039 |
| X2+0 μM | 45078 | 39202 |
| X3+0 μM | 71233 | 63523 |
| X1+0.4 μM | 2157 | 783 |
| X2+0.4 μM | 25899 | 18912 |
| X3+0.4 μM | 1084362 | 799673 |
| X1+0.6 μM | 653977 | 74312 |
| X2+0.6 μM | 124327 | 69906 |
| X3+0.6 μM | 32896 | 18395 |
| L1+0 μM | 44381 | 37890 |
| L2+0 μM | 155421 | 140748 |
| L3+0 μM | 167840 | 153608 |
| L1+0.4 μM | 229283 | 19229 |
| L2+0.4 μM | 22308 | 16653 |
| L3+0.4 μM | 544262 | 471173 |
| L1+0.6 μM | 227548 | 178091 |
| L2+0.6 μM | 263494 | 199258 |
| L3+0.6 μM | 293409 | 200992 |

‘+0 μM’, ‘+0.4 μM’, and ‘+0.6 μM’ imply that the samples were amplified with the blocking primer, X-BP2-DPO at the concentrations of 0 μM, 0.4 μM, and 0.6 μM in the PCRs. ‘X1, X2, and X3’, and ‘L1, L2, and L3’ are three different shrimp and oyster samples, respectively.

Table S4 Taxonomic composition of samples

| **Taxonomy** | **X1+0 μM** | **X1+0.4 μM** | **X1+0.6 μM** | **X2+0 μM** | **X2+0.4 μM** | **X2+0.6 μM** | **X3+0 μM** | **X3+0.4 μM** | **X3+0.6 μM** | **L1+0 μM** | **L1+0.4 μM** | **L1+0.6 μM** | **L2+0 μM** | **L2+0.4 μM** | **L2+0.6 μM** | **L3+0 μM** | **L3+0.4 μM** | **L3+0.6 μM** |
| --- | --- | --- | --- | --- | --- | --- | --- | --- | --- | --- | --- | --- | --- | --- | --- | --- | --- | --- |
| Poales | 0 | 0 | 0 | 0 | 9 | 0 | 0 | 0 | 0 | 0 | 0 | 0 | 0 | 0 | 0 | 0 | 0 | 0 |
| Magnoliophyta | 0 | 0 | 17 | 0 | 12 | 0 | 0 | 15 | 8 | 0 | 0 | 0 | 0 | 0 | 27 | 0 | 0 | 5 |
| Sphaeropleales | 0 | 0 | 16 | 0 | 0 | 0 | 0 | 0 | 0 | 0 | 0 | 23 | 0 | 0 | 0 | 0 | 0 | 0 |
| Sphaeropleales | 0 | 0 | 13 | 0 | 0 | 0 | 0 | 0 | 0 | 0 | 0 | 0 | 0 | 0 | 0 | 0 | 0 | 0 |
| uncultured eukaryote | 0 | 0 | 62 | 0 | 0 | 0 | 0 | 0 | 0 | 0 | 0 | 44 | 0 | 0 | 58 | 0 | 0 | 0 |
| Chlorophyceae | 0 | 0 | 800 | 0 | 0 | 59 | 0 | 0 | 24 | 0 | 0 | 631 | 0 | 0 | 803 | 0 | 9 | 153 |
| Trebouxiophyceae | 0 | 0 | 1087 | 0 | 0 | 183 | 0 | 0 | 44 | 0 | 0 | 11709 | 0 | 0 | 938 | 0 | 0 | 326 |
| Chlorellales | 0 | 0 | 0 | 0 | 0 | 0 | 0 | 0 | 0 | 0 | 0 | 39 | 0 | 0 | 0 | 0 | 0 | 0 |
| Trebouxiophyceae | 0 | 0 | 236 | 0 | 0 | 0 | 0 | 0 | 0 | 0 | 0 | 154 | 0 | 0 | 359 | 0 | 0 | 52 |
| Chlorophyta | 0 | 0 | 194 | 0 | 0 | 0 | 0 | 0 | 6 | 0 | 0 | 138 | 0 | 0 | 235 | 0 | 0 | 12 |
| Chloroplastida | 0 | 0 | 11 | 0 | 0 | 0 | 0 | 0 | 0 | 0 | 0 | 7 | 0 | 0 | 18 | 0 | 0 | 0 |
| Chroomonas | 0 | 0 | 359 | 0 | 16 | 26 | 14 | 0 | 7 | 0 | 0 | 34 | 0 | 0 | 140 | 0 | 0 | 30 |
| Guillardia | 0 | 0 | 367 | 0 | 0 | 35 | 0 | 0 | 0 | 0 | 0 | 75 | 0 | 0 | 263 | 0 | 2 | 45 |
| Rhodomonas | 0 | 0 | 5 | 0 | 0 | 0 | 0 | 0 | 0 | 0 | 0 | 0 | 0 | 0 | 0 | 0 | 0 | 0 |
| Cryptomonadales | 0 | 0 | 6725 | 39 | 36 | 231 | 33 | 58 | 41 | 0 | 0 | 433 | 8 | 0 | 3135 | 0 | 50 | 297 |
| uncultured eukaryote | 0 | 0 | 505 | 0 | 0 | 25 | 0 | 0 | 10 | 0 | 0 | 117 | 0 | 0 | 392 | 0 | 5 | 84 |
| Euglena tristella | 0 | 0 | 0 | 0 | 0 | 0 | 0 | 0 | 0 | 0 | 0 | 0 | 0 | 0 | 19 | 0 | 0 | 0 |
| Euglena | 0 | 0 | 42 | 0 | 0 | 6 | 0 | 0 | 0 | 0 | 0 | 24 | 0 | 0 | 92 | 0 | 0 | 19 |
| Metakinetoplastina | 0 | 0 | 0 | 0 | 0 | 0 | 0 | 0 | 0 | 0 | 0 | 0 | 0 | 0 | 5 | 0 | 0 | 0 |
| Learamoeba | 0 | 0 | 5 | 0 | 0 | 0 | 0 | 0 | 0 | 0 | 0 | 0 | 0 | 0 | 5 | 0 | 0 | 0 |
| Tetramitia | 0 | 0 | 0 | 0 | 0 | 0 | 0 | 0 | 0 | 0 | 0 | 0 | 0 | 0 | 6 | 0 | 0 | 0 |
| Reclinomonas americana | 0 | 0 | 32 | 0 | 0 | 3 | 0 | 0 | 4 | 0 | 0 | 5 | 0 | 0 | 43 | 0 | 0 | 7 |
| Pavlovophyceae | 0 | 0 | 0 | 0 | 0 | 0 | 0 | 0 | 5 | 0 | 0 | 0 | 0 | 0 | 7 | 0 | 0 | 0 |
| uncultured choanoflagellate | 0 | 0 | 5 | 0 | 0 | 6 | 0 | 0 | 0 | 0 | 0 | 26 | 0 | 0 | 22 | 0 | 0 | 22 |
| Penaeus monodon | 38770 | 680 | 16 | 36049 | 17612 | 0 | 61097 | 68894 | 0 | 413 | 457 | 7 | 4750 | 228 | 22 | 1697 | 6344 | 0 |
| Calanoida | 0 | 0 | 0 | 0 | 0 | 0 | 0 | 0 | 0 | 0 | 0 | 0 | 0 | 0 | 13 | 0 | 0 | 0 |
| Cyclopoida | 0 | 0 | 39 | 0 | 0 | 12 | 0 | 6 | 65 | 0 | 0 | 0 | 5 | 0 | 441 | 7 | 39 | 24 |
| Copepoda | 0 | 0 | 0 | 0 | 0 | 0 | 0 | 0 | 0 | 0 | 0 | 0 | 0 | 0 | 2 | 0 | 0 | 0 |
| Sessilia | 0 | 0 | 7 | 0 | 0 | 6 | 0 | 0 | 9 | 0 | 0 | 0 | 0 | 0 | 242 | 0 | 10 | 8 |
| Crustacea | 0 | 0 | 8 | 0 | 0 | 0 | 0 | 0 | 0 | 0 | 0 | 0 | 0 | 0 | 23 | 0 | 0 | 0 |
| Neoptera | 0 | 0 | 0 | 0 | 0 | 8 | 0 | 0 | 0 | 0 | 0 | 0 | 0 | 0 | 21 | 0 | 7 | 0 |
| Arthropoda | 0 | 0 | 0 | 0 | 0 | 0 | 4 | 0 | 0 | 0 | 0 | 0 | 0 | 0 | 0 | 0 | 0 | 0 |
| Teleostei | 0 | 0 | 0 | 0 | 0 | 0 | 0 | 16 | 0 | 0 | 0 | 0 | 0 | 0 | 0 | 0 | 13 | 0 |
| Mammalia | 0 | 0 | 7 | 12 | 0 | 416 | 0 | 0 | 0 | 12 | 0 | 0 | 0 | 0 | 0 | 0 | 0 | 0 |
| Chaetonotida | 0 | 0 | 5 | 0 | 0 | 0 | 0 | 0 | 0 | 0 | 0 | 0 | 0 | 0 | 9 | 0 | 0 | 0 |
| Chaetonotida | 0 | 0 | 12 | 0 | 0 | 0 | 0 | 0 | 0 | 0 | 0 | 0 | 0 | 0 | 0 | 0 | 0 | 0 |
| Veneroida | 0 | 0 | 0 | 0 | 0 | 0 | 0 | 0 | 0 | 0 | 0 | 0 | 0 | 0 | 0 | 2 | 0 | 0 |
| Ostreoida | 1083 | 103 | 19434 | 1309 | 668 | 65424 | 1637 | 4548 | 17014 | 37423 | 18707 | 156600 | 122162 | 16175 | 130137 | 151780 | 464001 | 193431 |
| Mollusca | 0 | 0 | 0 | 0 | 0 | 0 | 0 | 0 | 0 | 9 | 0 | 0 | 0 | 0 | 0 | 24 | 0 | 0 |
| Spirinia elongata | 0 | 0 | 0 | 0 | 0 | 0 | 0 | 0 | 0 | 0 | 0 | 0 | 0 | 0 | 0 | 0 | 4 | 0 |
| Ploimida | 0 | 0 | 16 | 0 | 0 | 15 | 0 | 0 | 10 | 0 | 0 | 0 | 0 | 0 | 863 | 0 | 12 | 21 |
| Bilateria | 0 | 0 | 15 | 0 | 0 | 0 | 0 | 0 | 6 | 0 | 0 | 0 | 0 | 0 | 33 | 6 | 4 | 0 |
| Eumetazoa | 0 | 0 | 9 | 0 | 0 | 0 | 0 | 0 | 0 | 0 | 0 | 0 | 0 | 0 | 5 | 0 | 0 | 0 |
| Metazoa | 0 | 0 | 444 | 0 | 0 | 102 | 4 | 0 | 35 | 0 | 0 | 243 | 0 | 0 | 479 | 0 | 0 | 264 |
| Holozoa | 0 | 0 | 169 | 0 | 0 | 42 | 0 | 0 | 11 | 0 | 0 | 117 | 0 | 0 | 249 | 0 | 0 | 129 |
| uncultured eukaryote | 0 | 0 | 24 | 0 | 0 | 0 | 0 | 0 | 0 | 0 | 0 | 0 | 0 | 0 | 0 | 0 | 0 | 0 |
| Incertae Sedis | 0 | 0 | 10 | 0 | 0 | 0 | 0 | 0 | 0 | 0 | 0 | 0 | 0 | 0 | 9 | 0 | 0 | 0 |
| Catenophlyctis variabilis | 0 | 0 | 3 | 0 | 0 | 0 | 0 | 0 | 0 | 0 | 0 | 0 | 0 | 0 | 8 | 0 | 0 | 4 |
| uncultured Chytridiomycota | 0 | 0 | 8 | 0 | 0 | 0 | 0 | 0 | 0 | 0 | 0 | 0 | 0 | 0 | 10 | 0 | 0 | 0 |
| Rhizophydium sp. JEL317 | 0 | 0 | 0 | 0 | 0 | 0 | 0 | 0 | 0 | 0 | 0 | 0 | 0 | 0 | 3 | 0 | 0 | 0 |
| uncultured Chytridiomycota | 0 | 0 | 0 | 0 | 0 | 9 | 0 | 0 | 0 | 0 | 0 | 32 | 0 | 0 | 0 | 0 | 0 | 0 |
| uncultured | 0 | 0 | 9 | 0 | 0 | 0 | 0 | 0 | 0 | 0 | 0 | 0 | 0 | 0 | 0 | 0 | 0 | 0 |
| Chytridiomycetes | 0 | 0 | 0 | 0 | 0 | 5 | 0 | 0 | 0 | 0 | 0 | 6 | 0 | 0 | 0 | 0 | 0 | 10 |
| Dothideomycetes | 0 | 0 | 0 | 0 | 0 | 0 | 0 | 0 | 0 | 0 | 0 | 0 | 0 | 0 | 18 | 0 | 0 | 0 |
| Pezizomycotina | 0 | 0 | 11 | 0 | 0 | 0 | 0 | 0 | 0 | 0 | 0 | 0 | 0 | 0 | 0 | 0 | 0 | 0 |
| Saccharomyces cerevisiae W303 | 0 | 0 | 0 | 0 | 0 | 0 | 0 | 0 | 0 | 0 | 0 | 0 | 0 | 0 | 0 | 0 | 0 | 8 |
| Saccharomycetales | 0 | 0 | 0 | 0 | 0 | 0 | 0 | 0 | 0 | 0 | 0 | 5 | 0 | 0 | 0 | 0 | 0 | 0 |
| Dikarya | 0 | 0 | 68 | 0 | 0 | 0 | 0 | 0 | 0 | 0 | 0 | 20 | 0 | 0 | 132 | 0 | 0 | 15 |
| uncultured eukaryote | 0 | 0 | 0 | 0 | 0 | 0 | 0 | 0 | 0 | 0 | 0 | 0 | 0 | 0 | 16 | 0 | 0 | 0 |
| Fungi | 0 | 0 | 595 | 0 | 0 | 50 | 0 | 0 | 10 | 0 | 0 | 100 | 0 | 0 | 249 | 0 | 0 | 88 |
| Nucletmycea | 0 | 0 | 3885 | 0 | 0 | 449 | 0 | 0 | 187 | 0 | 0 | 1181 | 0 | 0 | 3792 | 0 | 0 | 1232 |
| Opisthokonta | 26 | 0 | 98 | 28 | 19 | 18 | 31 | 117 | 8 | 0 | 0 | 64 | 11 | 3 | 240 | 0 | 18 | 69 |
| Paramecium | 0 | 0 | 19 | 0 | 0 | 4 | 0 | 0 | 0 | 0 | 0 | 10 | 0 | 0 | 22 | 0 | 0 | 0 |
| Oligohymenophorea | 0 | 0 | 33 | 0 | 0 | 0 | 0 | 0 | 0 | 0 | 0 | 0 | 0 | 0 | 31 | 0 | 0 | 0 |
| Coleps hirtus hirtus | 0 | 0 | 6 | 0 | 0 | 0 | 0 | 0 | 0 | 0 | 0 | 0 | 0 | 0 | 0 | 0 | 0 | 0 |
| Conthreep | 0 | 0 | 635 | 0 | 0 | 24 | 0 | 0 | 0 | 0 | 0 | 117 | 0 | 0 | 587 | 0 | 0 | 46 |
| Tintinnopsis sp. HCB-2005 | 0 | 0 | 79 | 0 | 0 | 8 | 0 | 0 | 9 | 0 | 0 | 16 | 0 | 0 | 74 | 0 | 0 | 6 |
| uncultured Halteria | 22 | 0 | 3878 | 23 | 81 | 485 | 203 | 34 | 57 | 0 | 0 | 585 | 19 | 0 | 2179 | 20 | 43 | 383 |
| Hypotrichia | 0 | 0 | 1004 | 0 | 0 | 18 | 10 | 12 | 0 | 0 | 0 | 77 | 0 | 0 | 511 | 0 | 0 | 43 |
| Oligotrichia | 0 | 0 | 1958 | 13 | 25 | 159 | 31 | 23 | 67 | 0 | 0 | 400 | 8 | 0 | 2305 | 10 | 36 | 358 |
| Intramacronucleata | 0 | 0 | 203 | 0 | 0 | 0 | 0 | 0 | 0 | 0 | 0 | 0 | 0 | 0 | 528 | 0 | 0 | 0 |
| Dinophyceae | 0 | 0 | 6 | 0 | 0 | 0 | 0 | 0 | 0 | 0 | 0 | 0 | 0 | 0 | 12 | 0 | 0 | 0 |
| uncultured alveolate | 0 | 0 | 6 | 0 | 0 | 0 | 0 | 0 | 0 | 0 | 0 | 0 | 0 | 0 | 11 | 0 | 0 | 0 |
| uncultured eukaryote | 0 | 0 | 443 | 0 | 0 | 27 | 6 | 0 | 12 | 0 | 0 | 92 | 0 | 0 | 3077 | 0 | 29 | 75 |
| Alveolata | 0 | 0 | 1486 | 0 | 0 | 73 | 7 | 4 | 14 | 0 | 0 | 318 | 7 | 0 | 4467 | 0 | 48 | 162 |
| Cercomonas | 0 | 0 | 2 | 0 | 0 | 0 | 0 | 0 | 0 | 0 | 0 | 0 | 0 | 0 | 0 | 0 | 0 | 0 |
| uncultured cercozoan | 0 | 0 | 0 | 7 | 0 | 59 | 17 | 59 | 22 | 0 | 0 | 126 | 0 | 0 | 117 | 0 | 0 | 172 |
| uncultured eukaryote | 0 | 0 | 145 | 0 | 0 | 15 | 0 | 0 | 0 | 0 | 0 | 42 | 0 | 0 | 99 | 0 | 2 | 34 |
| Adriamonas peritocrescens | 0 | 0 | 681 | 0 | 0 | 21 | 0 | 0 | 33 | 0 | 0 | 62 | 0 | 0 | 180 | 0 | 0 | 51 |
| Bicosoeca | 0 | 0 | 46 | 0 | 0 | 0 | 0 | 0 | 0 | 0 | 0 | 0 | 0 | 0 | 117 | 0 | 0 | 10 |
| uncultured eukaryote | 0 | 0 | 288 | 0 | 0 | 13 | 0 | 0 | 0 | 0 | 0 | 34 | 0 | 0 | 199 | 0 | 0 | 34 |
| Bicosoecida | 0 | 0 | 92 | 0 | 0 | 0 | 0 | 0 | 0 | 0 | 0 | 16 | 0 | 0 | 55 | 0 | 0 | 25 |
| Gerris buenoi | 0 | 0 | 441 | 0 | 0 | 34 | 0 | 0 | 19 | 0 | 0 | 58 | 0 | 0 | 252 | 0 | 5 | 42 |
| Chromulinales | 0 | 0 | 95 | 0 | 0 | 0 | 0 | 0 | 0 | 0 | 0 | 0 | 0 | 0 | 0 | 0 | 0 | 0 |
| LG01-09 | 0 | 0 | 46 | 0 | 0 | 0 | 0 | 0 | 0 | 0 | 0 | 0 | 0 | 0 | 33 | 0 | 0 | 0 |
| Glycine max (soybean) | 0 | 0 | 0 | 0 | 0 | 0 | 0 | 0 | 0 | 4 | 0 | 0 | 0 | 0 | 0 | 0 | 0 | 0 |
| Ochromonas | 0 | 0 | 191 | 0 | 0 | 11 | 0 | 0 | 0 | 0 | 0 | 20 | 0 | 0 | 114 | 0 | 0 | 13 |
| uncultured eukaryote | 0 | 0 | 380 | 0 | 0 | 42 | 11 | 0 | 0 | 0 | 0 | 38 | 0 | 0 | 455 | 0 | 0 | 28 |
| Ochromonas | 0 | 0 | 830 | 17 | 0 | 24 | 0 | 0 | 54 | 0 | 0 | 80 | 0 | 0 | 490 | 0 | 0 | 68 |
| Chrysophyceae | 0 | 0 | 513 | 0 | 0 | 0 | 3 | 0 | 0 | 0 | 0 | 54 | 0 | 0 | 376 | 0 | 0 | 31 |
| Bacillariophyceae | 0 | 0 | 19 | 0 | 0 | 0 | 0 | 0 | 0 | 0 | 0 | 0 | 0 | 0 | 0 | 0 | 0 | 0 |
| Bacillariophyceae | 0 | 0 | 1195 | 8 | 0 | 51 | 0 | 13 | 30 | 0 | 0 | 100 | 0 | 0 | 694 | 0 | 15 | 82 |
| Chaetoceros | 0 | 0 | 0 | 0 | 0 | 0 | 0 | 0 | 0 | 0 | 0 | 0 | 0 | 0 | 6 | 0 | 0 | 0 |
| Cyclotella choctawhatcheeana | 10 | 0 | 6160 | 28 | 36 | 559 | 14 | 62 | 162 | 9 | 0 | 1136 | 15 | 0 | 22256 | 17 | 185 | 1025 |
| Thalassiosira | 0 | 0 | 9 | 0 | 0 | 0 | 0 | 0 | 0 | 0 | 0 | 0 | 0 | 0 | 11 | 0 | 0 | 0 |
| Bacillariophytina | 0 | 0 | 20 | 0 | 0 | 0 | 0 | 0 | 0 | 0 | 0 | 0 | 0 | 0 | 34 | 0 | 0 | 0 |
| uncultured dictyochophyte | 0 | 0 | 30 | 0 | 0 | 0 | 0 | 0 | 2 | 0 | 0 | 5 | 0 | 0 | 27 | 0 | 0 | 7 |
| Eustigmatales | 0 | 0 | 37 | 0 | 0 | 0 | 0 | 0 | 0 | 0 | 0 | 0 | 0 | 0 | 38 | 0 | 0 | 0 |
| Ochrophyta | 0 | 0 | 2539 | 0 | 0 | 0 | 0 | 0 | 0 | 0 | 0 | 17 | 0 | 0 | 273 | 0 | 19 | 13 |
| Peronosporomycetes | 0 | 0 | 28 | 0 | 0 | 27 | 0 | 20 | 9 | 0 | 0 | 62 | 0 | 0 | 49 | 0 | 0 | 81 |
| uncultured stramenopile | 0 | 0 | 0 | 0 | 0 | 0 | 0 | 0 | 0 | 0 | 0 | 0 | 0 | 0 | 24 | 0 | 0 | 0 |
| Placididea | 0 | 0 | 174 | 0 | 0 | 23 | 5 | 6 | 4 | 0 | 0 | 53 | 0 | 0 | 0 | 0 | 0 | 60 |
| Stramenopiles | 0 | 0 | 1836 | 22 | 0 | 130 | 13 | 50 | 80 | 0 | 0 | 324 | 0 | 0 | 3598 | 0 | 35 | 221 |
| SAR | 0 | 0 | 4483 | 0 | 0 | 77 | 0 | 45 | 34 | 0 | 0 | 207 | 0 | 0 | 1283 | 0 | 20 | 164 |
| Eukaryota | 128 | 0 | 8903 | 1647 | 398 | 892 | 383 | 725691 | 283 | 20 | 65 | 2208 | 13763 | 247 | 11091 | 45 | 218 | 1403 |

‘+0 μM’, ‘+0.4 μM’, and ‘+0.6 μM’ imply that the samples were amplified with the blocking primer, X-BP2-DPO at the concentrations of 0 μM, 0.4 μM, and 0.6 μM in the PCRs. ‘X1, X2, and X3′, and ‘L1, L2, and L3′ are three different shrimp and oyster samples, respectively
